# LncRNA Snhg6 regulates the differentiation of MDSCs by regulating the ubiquitination of EZH2

**DOI:** 10.1186/s13045-021-01212-0

**Published:** 2021-11-18

**Authors:** Wei Lu, Fenghua Cao, Lili Feng, Ge Song, Yi Chang, Ying Chu, Zhihong Chen, Bo Shen, Huaxi Xu, Shengjun Wang, Jie Ma

**Affiliations:** 1 Department of Immunology, Jiangsu Key Laboratory of Laboratory Medicine, School of Medicine, Jiangsu University, Zhenjiang, 212013 China; 2 Department of Laboratory Medicine, Liyuan Hospital, Tongji Medical College, Huazhong University of Science and Technology, Hubei, China; 3 Zhenjiang Hospital of Traditional Chinese and Western Medicine, Zhenjiang, 212000 China; 4 Department of Gastrointestinal Surgery, Affiliated Renmin Hospital of Jiangsu University: Zhenjiang First People’s Hospital, Zhenjiang, 212002 Jiangsu China; 5 The Affiliated Cancer Hospital of Nanjing Medical University, Jiangsu Cancer Hospital and Jiangsu Institute of Cancer Research, Nanjing, 210009 China

**Keywords:** MDSCs, lncRNA Snhg6, EZH2, ubiquitination, Differentiation

## Abstract

Myeloid-derived suppressor cells (MDSCs) are derived from bone marrow progenitor cells commonly, which is a heterogeneous cell group composed of immature granulocytes, dendritic cells, macrophages and early undifferentiated bone marrow precursor cells. Its differentiation and immunosuppressive function are regulated by complex network signals, but the specific regulation mechanisms are not yet fully understood. In this study, we found that in mouse of Lewis lung cancer xenograft, long non-coding RNA Snhg6 (lncRNA Snhg6) was highly expressed in tumor-derived MDSCs compared with spleen-derived MDSCs. LncRNA Snhg6 facilitated the differentiation of CD11b^+^ Ly6G^−^ Ly6C^high^ monocytic MDSCs (Mo-MDSCs) rather than CD11b^+^ Ly6G^+^ Ly6C^low^ polymorphonuclear MDSCs (PMN-MDSCs), but did not affect the immunosuppressive function of MDSCs. Notably, lncRNA Snhg6 could inhibit the expression of EZH2 by ubiquitination pathway at protein level rather than mRNA level during the differentiation of mouse bone marrow cells into MDSCs in vitro. EZH2 may be an important factor in the regulation of lncRNA Snhg6 to promote the differentiation of Mo-MDSCs. So what we found may provide new ideas and targets for anti-tumor immunotherapy targeting MDSCs.

**Supplementary Information:**

The online version contains supplementary material available at 10.1186/s13045-021-01212-0.

## To the Editor

MDSCs are not a single defined cell population in myeloid cells, but a mixture of a large number of granulocytes, macrophages, and dendritic cells that are hindered in differentiation and maturation. The phenotypic identification of MDSCs is extremely complicated, MDSCs mainly co-express CD11b and Gr-1, which mainly including PMN-MDSCs (CD11b^+^ Ly6G^+^ Ly6C^low^) and Mo-MDSCs (CD11b^+^ Ly6G^−^ Ly6C^high^) in mice. They usually perform immunosuppressive function in different ways [1, 2]. Increasing evidences show that lncRNAs play an important role in the establishment of immune cell lineage and immune response because of its complexity in regulation, self-composition and structure [3]. However, the relationship between lncRNAs and MDSCs has not attracted widespread attention.

**Fig. 1:**
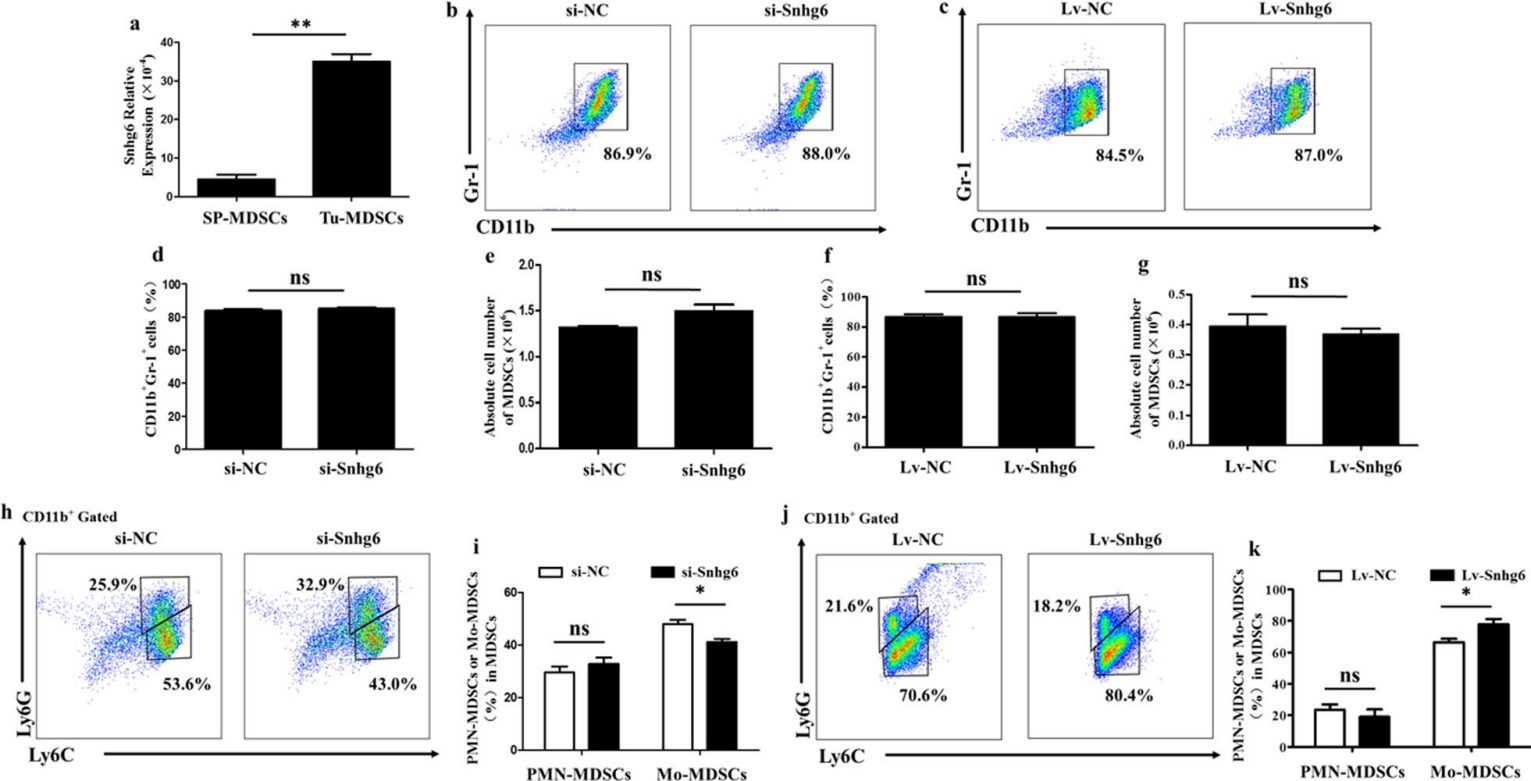
LncRNA Snhg6 promotes Mo-MDSCs but not affects the differentiation of PMN-MDSCs. **a** LncRNA Snhg6 expression in Tu-MDSCs compared with SP-MDSCs measured by qRT-PCR. **b, c** Typical flow cytometry of CD11b^+^ Gr-1^+^ MDSCs. **d–g** The percentage and absolute number of CD11b^+^ Gr-1^+^ MDSCs by statistical analyses. **h, i** Typical flow cytometry and the percentage of CD11b^+^ Ly6G^+^ Ly6C^low^ PMN-MDSCs and CD11b^+^ Ly6G^−^ Ly6C^high^ Mo-MDSCs after downregulating the expression of lncRNA Snhg6. **j, k** Typical flow cytometry and percentage of CD11b^+^ Ly6G^+^ Ly6C^low^ PMN-MDSCs and CD11b^+^ Ly6G^−^ Ly6C^high^ Mo-MDSCs after increasing the expression of lncRNA Snhg6. Each expression had three replicates, ns: no significance; * *p* < 0.05; ** *p* < 0.01

LncRNA Snhg6 is a novel lncRNA, which abnormally expresses in a variety of cancers [4–6]. By analyzing Arrarystar lncRNA microarray of Tu-MDSCs and SP-MDSCs (MDSCs derived from tumor tissue and spleen of mice with Lewis lung cancer xenograft, respectively), we finally chose lncRNA Snhg6, which is highly expressed in Tu-MDSCs, as the object of this study (Fig. 1a, Additional file 1: Fig. S1, Additional file 2: S2, Additional file 5: Table S1). To investigate the effects of lncRNA Snhg6 on MDSCs, we first transfected the specific siRNA (si-Snhg6) or overexpression lentivirus (Lv-Snhg6) of lncRNA Snhg6 in bone marrow cells and then induced MDSCs under the stimulation of GM-CSF and IL-6 (Additional file 3: Fig. S3, Additional file 6: Table S2.). The results revealed that the differentiation rate and absolute number of CD11b^+^ Gr-1^+^ MDSCs did not change significantly whether the expression of lncRNA Snhg6 was decreased or increased (Fig. 1b–g). Further studies showed that there was also no significant change in CD11b^+^ Ly6G^+^ Ly6C^low^ PMN-MDSCs, while the percentage of CD11b^+^ Ly6G^−^ Ly6C^high^ Mo-MDSCs was significantly reduced after lncRNA Snhg6–silencing (Fig. 1h, i). And overexpression of lncRNA Snhg6 increased the percentage of CD11b^+^ Ly6G^−^ Ly6C^high^ Mo-MDSCs (Fig. 1j, k). All of these indicated that lncRNA Snhg6 was involved in promoting the differentiation of Mo-MDSCs.

**Fig. 2:**
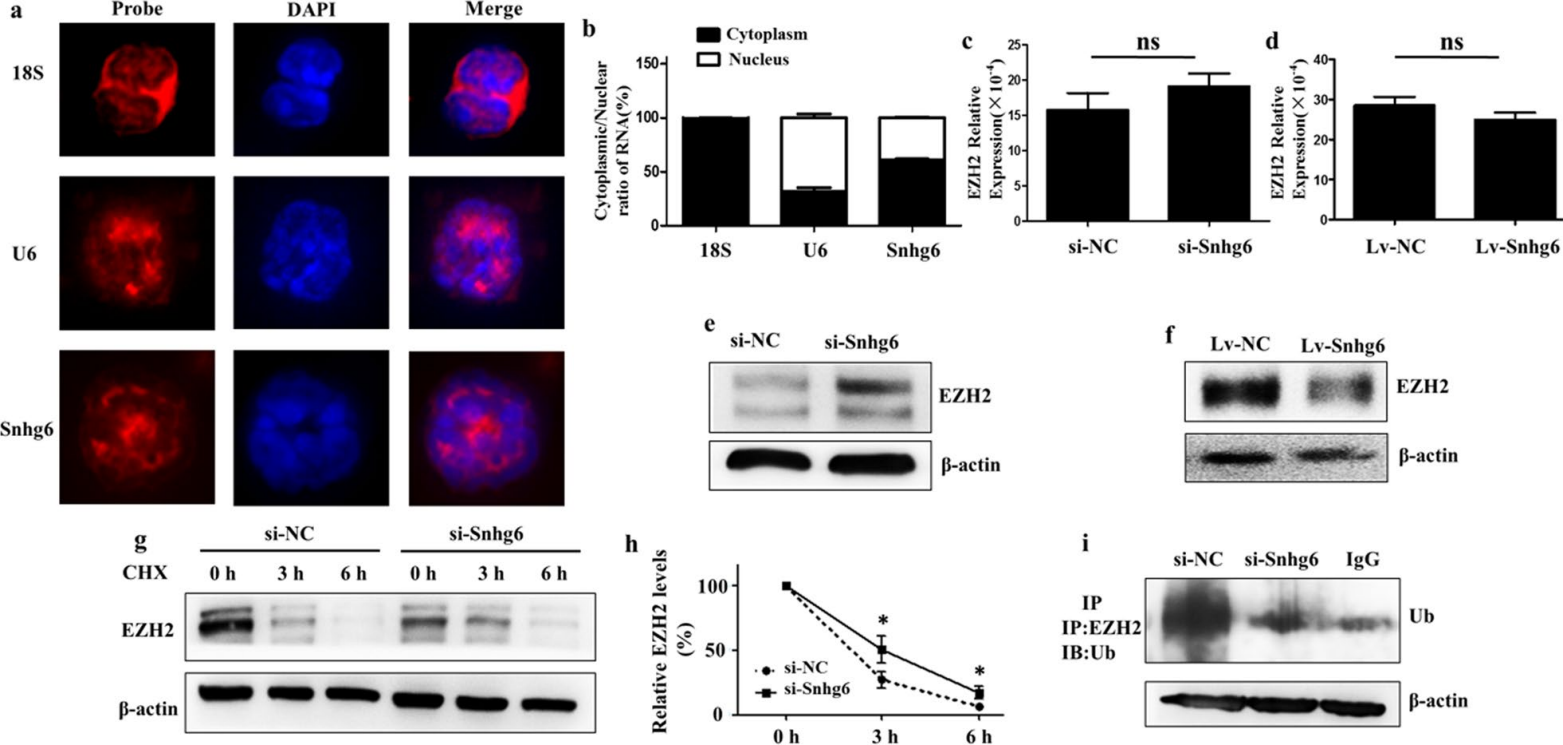
LncRNA Snhg6 reduces the stability of EZH2 protein. **a** The cellular localization of lncRNA Snhg6 was detected by Cy3-labeled lncRNA Snhg6 probe. Cy3-labeled 18S probe was used to indicate plasmid localization and Cy3-labeled U6 probe was used to indicate nuclear localization. DAPI was used to evaluate the cell nucleus. **b** Subcellular fractionation was isolated of MDSCs, and lncRNA Snhg6 localization was examined by qRT-PCR. 18S and U6 were used as cytoplasmic and nuclear indicators, respectively. **c, d** qRT-PCR were used to detect the expression of EZH2 at mRNA level. **e, f** Western blot were used to detect the expression of EZH2 at protein level. **g** Western blot were used to detect the expression of EZH2 with CHX (40 µg/ml) treated 0 h, 3 h and 6 h after transfecting si-Snhg6. **h** The statistical graph corresponding to the left. **i** RIP assays were used to investigate the ubiquitination of EZH2. Each expression had three replicates, ns: no significance; *p < 0.05; **p < 0.01

The specific mechanism by which lncRNAs play a regulatory role is often determined by their subcellular location [7]. So next we detected the cellular distribution of lncRNA Snhg6 in MDSCs. RNA Fluorescence in situ Hybridization (RNA-FISH) revealed that lncRNA Snhg6 was located in both the cytoplasm and the nucleus (Fig. 2a). In addition, we also measured the expression of lncRNA Snhg6 in nuclear and cytoplasmic fractions of MDSCs by qRT-PCR. The results were consistent with RNA-FISH, which further verified that lncRNA Snhg6 was mainly located in the cytoplasm of MDSCs (Fig. 2b). Histone methyltransferase Enhancer of Zeste homolog 2 (EZH2) is a histone methyltransferase catalyzing the methylation of histone H3 at lysine 27. The latest research showed that an inhibitor of EZH2 activity—GSK343 could significantly promote the differentiation of hematopoietic stem cells (HPCs) into MDSCs in the presence of granulocyte–macrophage colony-stimulating factors (GM-CSF) and interleukin-6 (IL-6) in vitro [8]. In addition, the involvement of lncRNA Snhg6 in regulating EZH2 has also been reported [6, 9]. So we speculate that lncRNA Snhg6 may regulate the differentiation of MDSCs through EZH2. The following experiment proved that lncRNA Snhg6 could regulate the expression of EZH2 at the post-transcriptional rather than transcriptional level (Fig. 2c–f, Additional file 5: Table S1). Subsequently, the protein expression of EZH2 was detected at 0 h, 3 h, and 6 h, respectively, after adding cycloheximide (CHX). The results revealed that the stability of EZH2 protein significantly improved after downregulating lncRNA Snhg6 (Fig. 2g, h). Further Immunoprecipitation (IP) testing showed that the ubiquitination level of EZH2 was obviously reduced as lncRNA Snhg6 decreased (Fig. 2i). These suggest that lncRNA Snhg6 was likely to regulate the stability of EZH2 through protein-ubiquitination degradation pathway in the differentiation process of MDSCs. Of course, protein could be degraded either by the ubiquitin proteasome or through the lysosomal pathway after the protein is ubiquitinated [10]. The specific degradation mechanism of EZH2 in our study remains to be further study.

The occurrence and development of tumors are inseparable from the tumor microenvironment with immunosuppressive characteristics, and the massive accumulation of immunosuppressive MDSCs in the tumor microenvironment is the main cause of tumor immune non-response. The previous experimental results in our laboratory confirmed that compared with SP-MDSCs, Tu-MDSCs had a stronger ability to inhibit CD4/CD8 T cells [11]. Therefore, we detected the inhibitory effect of MDSCs on CD4^+^ T proliferation and its immunosuppressive effector molecules arginase (Arg-1), nitric oxide (NO) and reactive oxygen species (ROS). All results showed that lncRNA Snhg6 did not participate in regulating the immunosuppressive function of MDSCs (Additional file 3: Fig. S3 and Additional file 4: Fig. S4, Additional file 5: Table S1).

In short, we found that lncRNA Snhg6 was involved in regulating the differentiation of MDSCs by reducing the protein stability of EZH2, but it did not affect the immunosuppressive function of MDSCs, which might provide a new perspective for the treatment of cancer.

## Supplementary Information

**Additional file 1: Fig. S1.** Association between lncRNA Snhg6 and MDSCs and its expression in lung adenocarcinoma. **a** The flow cytometry was used to evaluate the purity of MDSCs from different tissues by detecting the expression of two surface markers: Gr-1 and CD11b. **b** The clustering analysis of Arrarystar lncRNA microarray. **c** The raw intensity of lncRNA Snhg6 in Arrarystar lncRNA microarray detected by lncRNA probes. **d** The expression of lncRNA Snhg6 with 526 cancer and 59 normal samples in lung adenocarcinoma (LUAD) in starBase dataset. **e** Overall survival for lncRNA Snhg6 in LUAD cancer in starBase dataset.

**Additional file 2: Fig. S2.** The expression of lncRNA Snhg6 increased significantly in tumor microenvironment. **a** The differentiation percentage of CD11b^+^ Gr-1^+^ MDSCs after different percentage tumor cell conditioned medium (TCCM) treatment detected by FCM. 1640 : TCCM 1: 0 means that the volume ratio of 1640 complete culture fluid to TCCM was 1:0 (the rests are the same). **b** The expression of lncRNA Snhg6 after different percentage TCCM treatment detected by qRT-PCR. **c** The percentage of CD11b^+^Gr-1^+^ MDSCs induced by bone marrow cells with GM-CSF and IL-6 in vitro. Control: no treatment for bone marrow cells. GM-CSF+IL-6: Bone marrow cells induced by GM-CSF and IL-6. **d** The expression of lncRNA Snhg6 was upregulated after induced by GM-CSF and IL-6 in vitro. Each expression had three replicates, *p < 0.05.

**Additional file 3: Fig. S3.** The transfection efficiency of siRNA and overexpression lentivirus lncRNA Snhg6 under different conditions. **a** MDSCs were transfected with Cy3 labeled siRNA with red fluorescence. The transfection efficiency of siRNA was detected by fluorescence microscopy (×200). **b** During the induction of MDSCs by bone marrow cells, the expression of lncRNA Snhg6 was detected by qRT-PCR after transfecting siRNA Snhg6 001 (si-Snhg6 001), siRNA Snhg6 002 (si-Snhg6 002), siRNA Snhg6 003 (si-Snhg6 003) and negative control (si-NC). **c** During the induction of MDSCs by bone marrow cells, the expression of lncRNA Snhg6 was detected by qRT-PCR after transfecting overexpression lentivirus (Lv-Snhg6) and negative control (Lv-NC). **d** In Tu-MDSCs, qRT-PCR was preformed to measure the expression of lncRNA Snhg6 after transfecting with siRNA Snhg6 001 (si-Snhg6 001), siRNA Snhg6 002 (si-Snhg6 002), siRNA Snhg6 003 (si-Snhg6 003) and negative control (si-NC). Each expression had three replicates, ns: no significance; *p < 0.05; **p < 0.01; ***p < 0.001.

**Additional file 4: Fig. S4.** LncRNA Snhg6 was not involved in regulating the immunosuppressive function of MDSCs. **a** The activity of arginase (Arg-1) was measured by QuantiChrom Arginase Assay kit according to the instruction. **b** NO was measured with Griess Reagent System according to the instruction. **c** ROS was detected by flow cytometry after the oxidation-sensitive dye 2′,7′-dichlorofluorescin diacetate and PMA. **d** MDSCs were transfected with si-Snhg6 6 h, then the cells were harvested and co-cultured with CFSE labeled CD4^+^ T cells for 72 h under the stimulation of anti-CD3 mAb and anti-CD28 mAb. The proliferation of CD4^+^ T cells was measured by flow cytometry. Each expression had three replicates, ns: no significance.

**Additional file 5: Table S1.** Primers sequences.

**Additional file 6: Table S2.** siRNA Target Sequences of lncRNA Snhg6

## Data Availability

All supporting data are included in the manuscript and supplemental files.

## Abbreviations

MDSCs: Myeloid-derived suppressor cells
lncRNA Snhg6: LncRNA small nucleolar RNA host gene 6
Mo-MDSCs: Monocytic MDSCs
PMN-MDSCs: Polymorphonuclear MDSCs
Tu-MDSCs: MDSCs derived from tumor tissue of tumor-bearing mice
SP-MDSCs: MDSCs derived from spleen of tumor-bearing mice
si-Snhg6: SiRNA of lncRNA Snhg6
Lv-Snhg6: Overexpression lentivirus of lncRNA Snhg6
EZH2: Histone methyltransferase enhancer of Zeste homolog 2
Arg-1: Arginase
NO: Nitric oxide
ROS: Reactive oxygen species
