# Supplementary figures and images for: LncRNA Snhg6 regulates the differentiation of MDSCs by regulating the ubiquitination of EZH2
Source: J Hematol Oncol. 2021 Nov 18;14:196. doi: 10.1186/s13045-021-01212-0 (PMC8600792; doi:10.1186/s13045-021-01212-0)

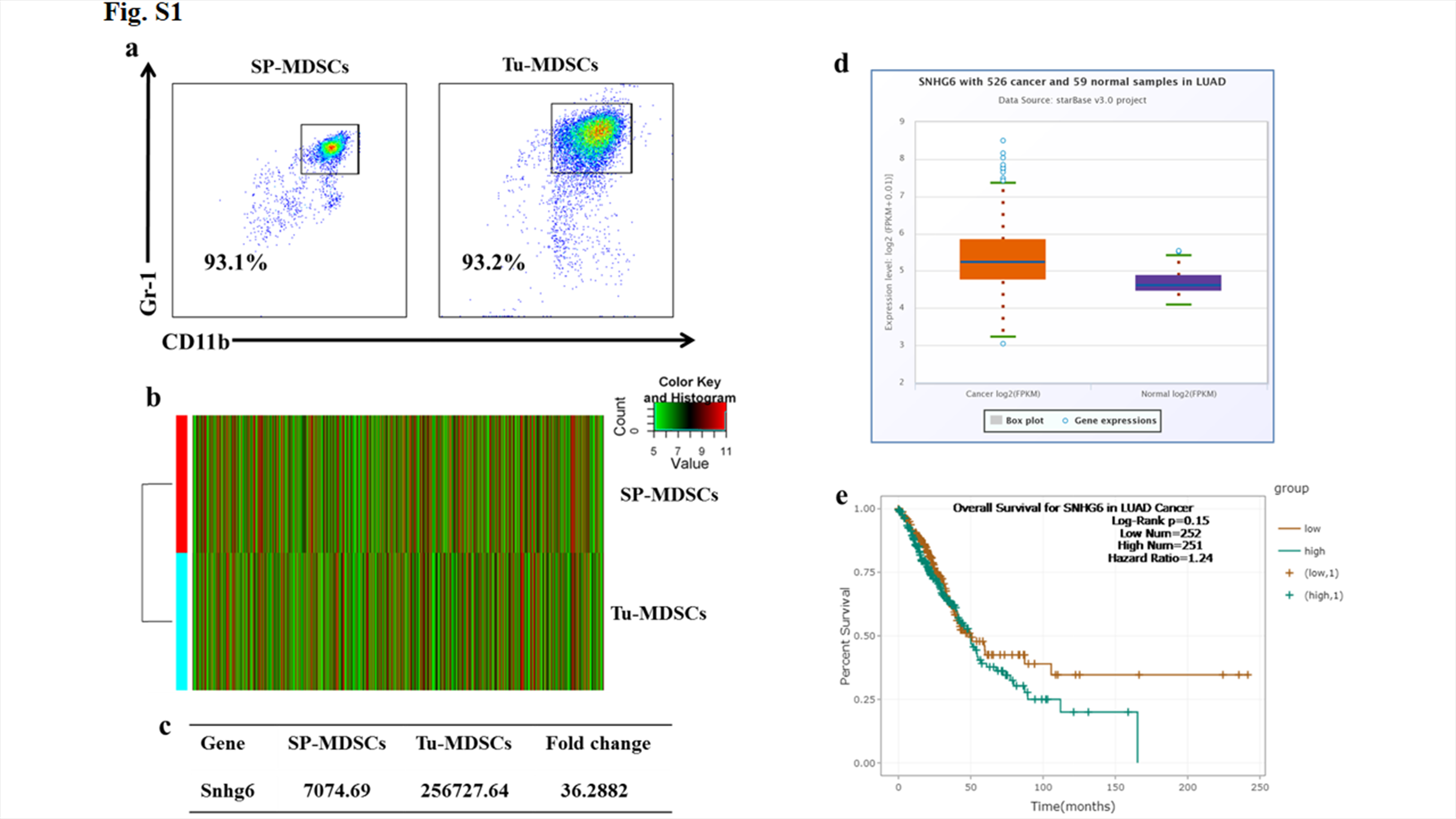

Supplement: Supplementary file 1 — Additional file 1: Fig. S1. Association between lncRNA Snhg6 and MDSCs and its expression in lung adenocarcinoma. a The flow cytometry was used to evaluate the purity of MDSCs from different tissues by detecting the expression of two surface markers: Gr-1 and CD11b. b The clustering analysis of Arrarystar lncRNA microarray. c The raw intensity of lncRNA Snhg6 in Arrarystar lncRNA microarray detected by lncRNA probes. d The expression of lncRNA Snhg6 with 526 cancer and 59 normal samples in lung adenocarcinoma (LUAD) in starBase dataset. e Overall survival for lncRNA Snhg6 in LUAD cancer in starBase dataset. [file 13045_2021_1212_MOESM1_ESM.tif]

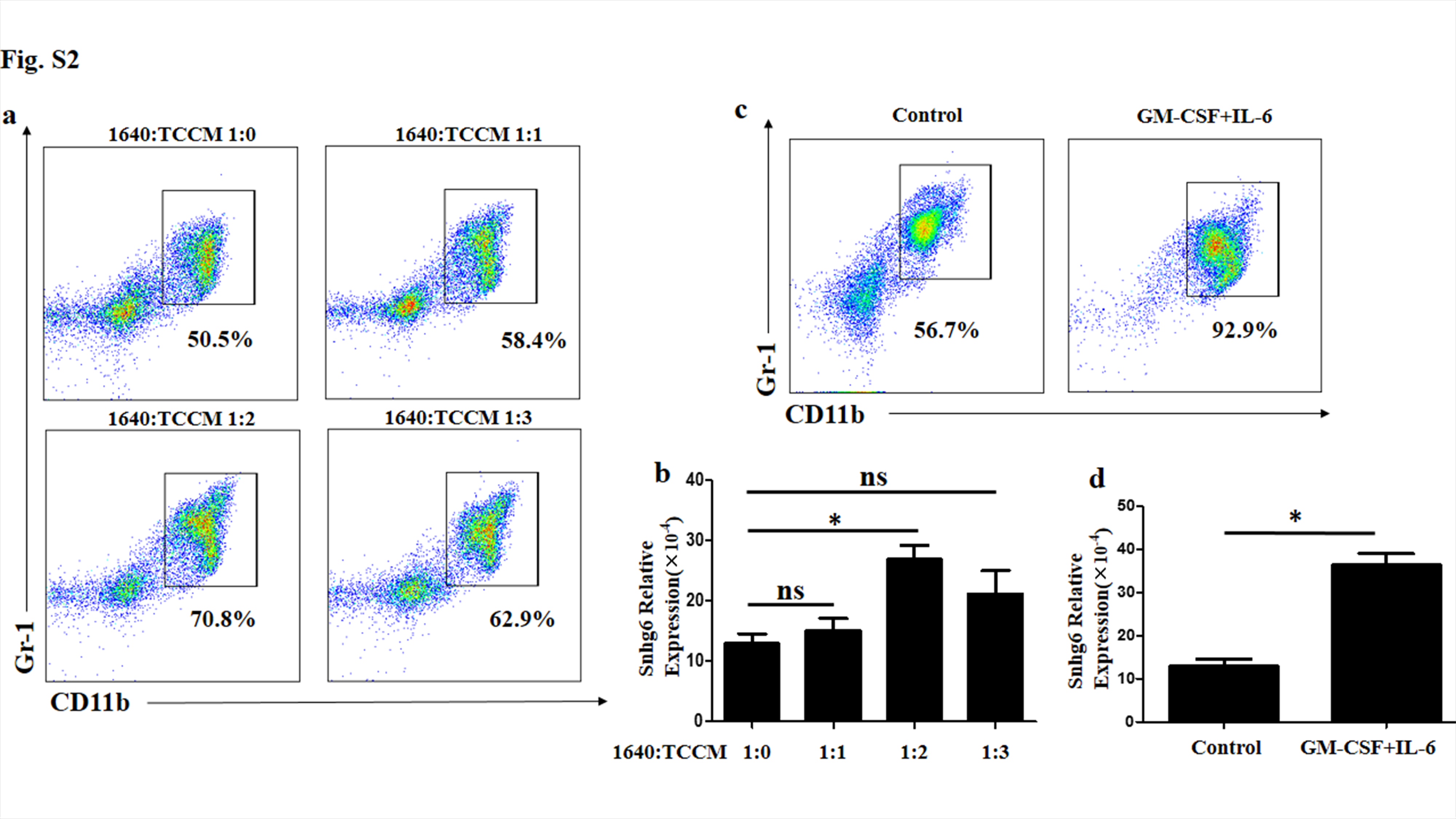

Supplement: Supplementary file 2 — Additional file 2: Fig. S2. The expression of lncRNA Snhg6 increased significantly in tumor microenvironment. a The differentiation percentage of CD11b+ Gr-1+ MDSCs after different percentage tumor cell conditioned medium (TCCM) treatment detected by FCM. 1640 : TCCM 1: 0 means that the volume ratio of 1640 complete culture fluid to TCCM was 1:0 (the rests are the same). b The expression of lncRNA Snhg6 after different percentage TCCM treatment detected by qRT-PCR. c The percentage of CD11b+Gr-1+ MDSCs induced by bone marrow cells with GM-CSF and IL-6 in vitro. Control: no treatment for bone marrow cells. GM-CSF+IL-6: Bone marrow cells induced by GM-CSF and IL-6. d The expression of lncRNA Snhg6 was upregulated after induced by GM-CSF and IL-6 in vitro. Each expression had three replicates, *p < 0.05. [file 13045_2021_1212_MOESM2_ESM.tif]

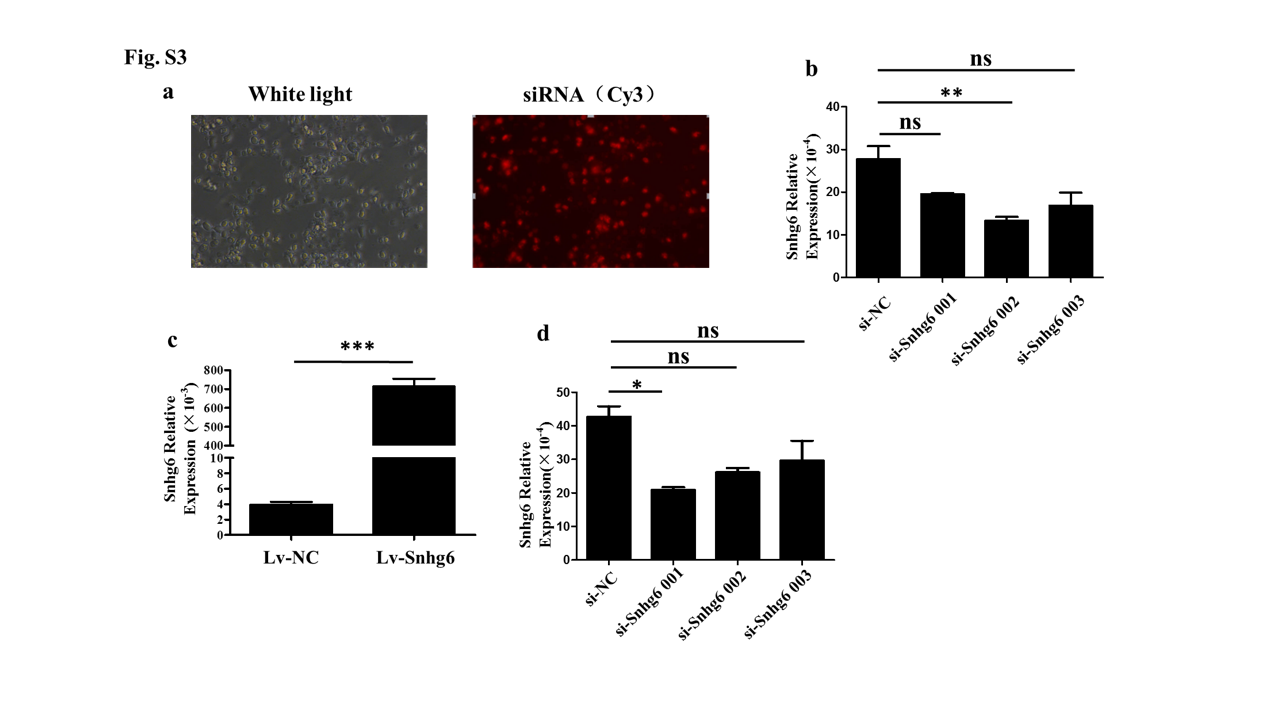

Supplement: Supplementary file 3 — Additional file 3: Fig. S3. The transfection efficiency of siRNA and overexpression lentivirus lncRNA Snhg6 under different conditions. a MDSCs were transfected with Cy3 labeled siRNA with red fluorescence. The transfection efficiency of siRNA was detected by fluorescence microscopy (×200). b During the induction of MDSCs by bone marrow cells, the expression of lncRNA Snhg6 was detected by qRT-PCR after transfecting siRNA Snhg6 001 (si-Snhg6 001), siRNA Snhg6 002 (si-Snhg6 002), siRNA Snhg6 003 (si-Snhg6 003) and negative control (si-NC). c During the induction of MDSCs by bone marrow cells, the expression of lncRNA Snhg6 was detected by qRT-PCR after transfecting overexpression lentivirus (Lv-Snhg6) and negative control (Lv-NC). d In Tu-MDSCs, qRT-PCR was preformed to measure the expression of lncRNA Snhg6 after transfecting with siRNA Snhg6 001 (si-Snhg6 001), siRNA Snhg6 002 (si-Snhg6 002), siRNA Snhg6 003 (si-Snhg6 003) and negative control (si-NC). Each expression had three replicates, ns: no significance; *p < 0.05; **p < 0.01; ***p < 0.001. [file 13045_2021_1212_MOESM3_ESM.tif]

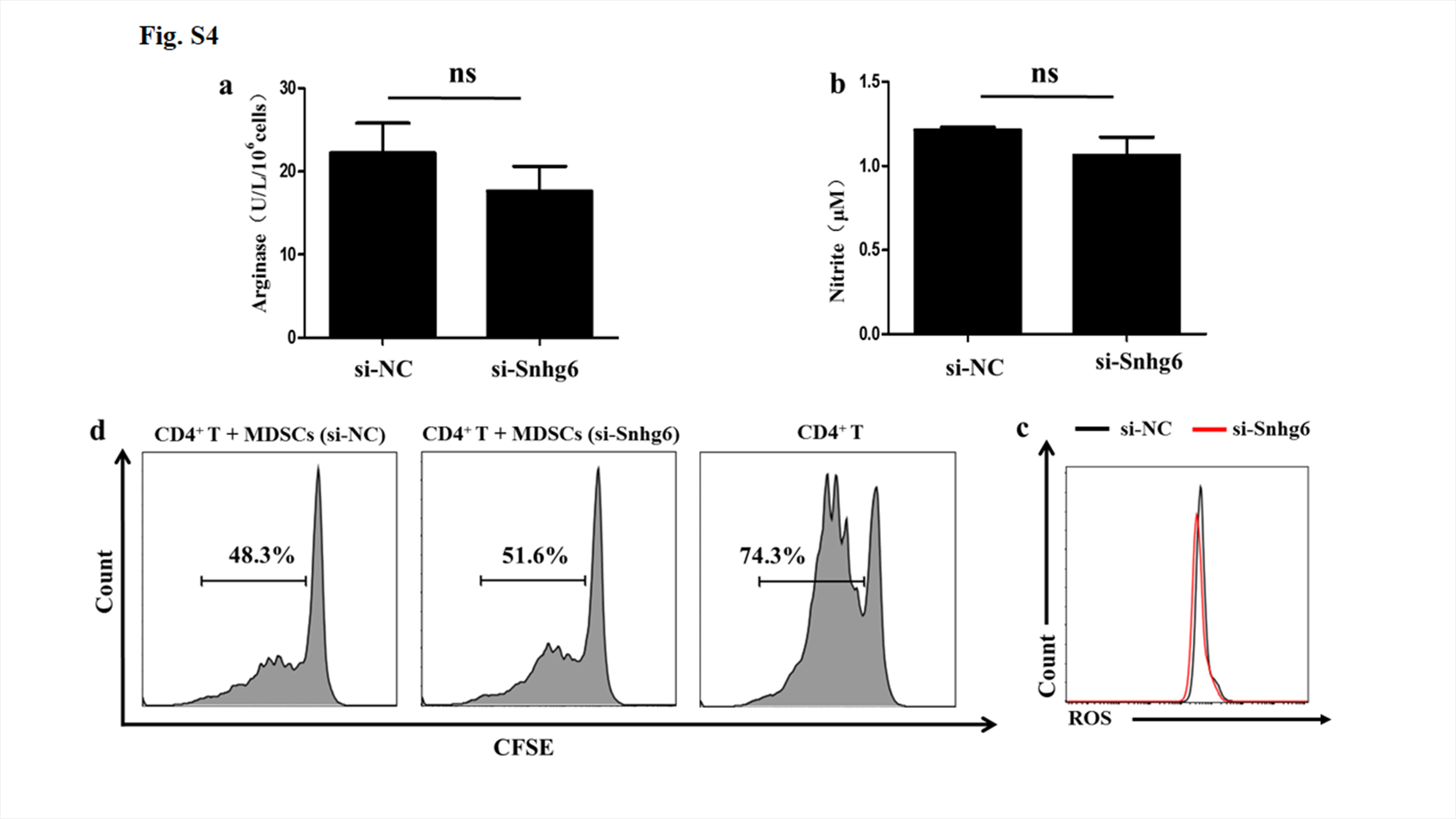

Supplement: Supplementary file 4 — Additional file 4: Fig. S4. LncRNA Snhg6 was not involved in regulating the immunosuppressive function of MDSCs. a The activity of arginase (Arg-1) was measured by QuantiChrom Arginase Assay kit according to the instruction. b NO was measured with Griess Reagent System according to the instruction. c ROS was detected by flow cytometry after the oxidation-sensitive dye 2′,7′-dichlorofluorescin diacetate and PMA. d MDSCs were transfected with si-Snhg6 6 h, then the cells were harvested and co-cultured with CFSE labeled CD4+ T cells for 72 h under the stimulation of anti-CD3 mAb and anti-CD28 mAb. The proliferation of CD4+ T cells was measured by flow cytometry. Each expression had three replicates, ns: no significance. [file 13045_2021_1212_MOESM4_ESM.tif]
