# Supplementary material for: LncRNA Snhg6 regulates the differentiation of MDSCs by regulating the ubiquitination of EZH2
Source: J Hematol Oncol. 2021 Nov 18;14:196. doi: 10.1186/s13045-021-01212-0 (PMC8600792; doi:10.1186/s13045-021-01212-0)
Supplement: Supplementary file 5 — Additional file 5: Table S1. Primers sequences. [file 13045_2021_1212_MOESM5_ESM.doc]

Additional file 5: Table S1. Primers sequences

| Gene name | Primer sequences |
| --- | --- |
| Snhg6 | Forward：5’-CAAAACCTGCTCATTTGAAGGTGA-3’  Reverse：5’- TTGGCAAATGTAGTCCTGGCA-3’ |
| β-actin | Forward：5’-GTGCTATGTTGCTCTAGACTTCG-3’  Reverse：5’-ATGCCACAGGATTCCATACC-3’ |
| EZH2 | Forward：5’-AGAGTGGAAGCAGCGGAGGATAC -3’ |
|  | Reverse：5’-CATTATAGGCACCGAGGCGACTG-3’ |
