# Supplementary material for: LncRNA Snhg6 regulates the differentiation of MDSCs by regulating the ubiquitination of EZH2
Source: J Hematol Oncol. 2021 Nov 18;14:196. doi: 10.1186/s13045-021-01212-0 (PMC8600792; doi:10.1186/s13045-021-01212-0)
Supplement: Supplementary file 6 — Additional file 6: Table S2. siRNA Target Sequences of lncRNA Snhg6 [file 13045_2021_1212_MOESM6_ESM.doc]

Additional file 6: Table S2. siRNA Target Sequences of lncRNA Snhg6

| Gene name | Target sequences |
| --- | --- |
| si-Snhg6 001 | 5’-CGTTTCCAGGTGCAACAAA-3’ |
| si-Snhg6 002 | 5’-GGACTACATTTGCCAATAA-3’ |
| si-Snhg6 003 | 5’-GCATGTAGGTGGCTGTAGT-3’ |
